# Supplementary material for: Radical triads, not pairs, may explain effects of hypomagnetic fields on neurogenesis
Source: PLoS Comput Biol. 2022 Sep 15;18(9):e1010519. doi: 10.1371/journal.pcbi.1010519 (PMC9514667; doi:10.1371/journal.pcbi.1010519)
Supplement: S1 Text — Table A. Hyperfine interaction tensors for FADH•. Fig A. Dependence of the singlet recombination yield ΦX on the flux density of the applied magnetic field for the FH•/O2•−/A•− triad system with instantaneous spin relaxation in O2•−. Fig B. Sensitivity of the hypomagnetic field effect of the FH•/O2•−/A•− triad system with instantaneous spin relaxation in O2•− to spin relaxation in the FH• and A•− radicals. Table B. Hypomagnetic field effects in FH•/O2•−/A•− triad system with instantaneous spin relaxation in O2•− calculated assuming absence or presence of spin relaxation in the FH• and A•− radicals (implemented as random-field relaxation). Fig C. The predicted hypomagnetic field effect for FH•/O2•−/A•− triad system with instantaneous spin relaxation in O2•− and variable spin relaxation in FH• and A•− radicals (random-field relaxation). Code listing A. Python code to simulate isotropic and anisotropic magnetic field effects in radical pairs using tools form QuTip. (PDF) [file pcbi.1010519.s001.pdf]

Supporting Information:

Radical triads, not pairs, may explain effects of hypomagnetic fields on neurogenesis

Jess Ramsay[†] and Daniel R. Kattnig[†,*]

† Living Systems Institute and Department of Physics, University of Exeter, Stocker Road, EX4 4QD, Exeter, U.K.

* Corresponding author: Daniel R. Kattnig, d.r.kattnig@exeter.ac.uk

Table A: Hyperfine interaction tensors for FADH$^\bullet$ [1].

$$\mathrm{N5} = \begin{pmatrix} -2.41 & -0.07 & -0.97 \\ -0.07 & -2.45 & 0.05 \\ -0.97 & 0.05 & 43.51 \end{pmatrix} \mathrm{MHz}$$

$$\mathrm{H5} = \begin{pmatrix} -2.39 & 1.87 & 0.51 \\ 1.87 & -40.64 & 0.03 \\ 0.51 & 0.03 & -27.86 \end{pmatrix} \mathrm{MHz}$$

$$\mathrm{N10} = \begin{pmatrix} 0.44 & 0.06 & 1.80 \\ 0.06 & -0.01 & -0.34 \\ 1.80 & -0.34 & 23.15 \end{pmatrix} \mathrm{MHz}$$

$$\mathrm{H6} = \begin{pmatrix} -2.25 & -1.74 & 0.09 \\ -1.74 & -4.74 & -0.08 \\ 0.09 & -0.08 & -6.07 \end{pmatrix} \mathrm{MHz}$$

$$\mathrm{H\beta 1} = \begin{pmatrix} 10.34 & -0.86 & -1.24 \\ -0.86 & 7.56 & 0.37 \\ -1.24 & 0.37 & 6.91 \end{pmatrix} \mathrm{MHz}$$

[1] J. Deviers, F. Cailliez, A. de la Lande, D. R. Kattnig, Anisotropic magnetic field effects in the re-oxidation of cryptochrome in the presence of scavenger radicals. *J. Chem. Phys.* **156**, 025101 (2021).

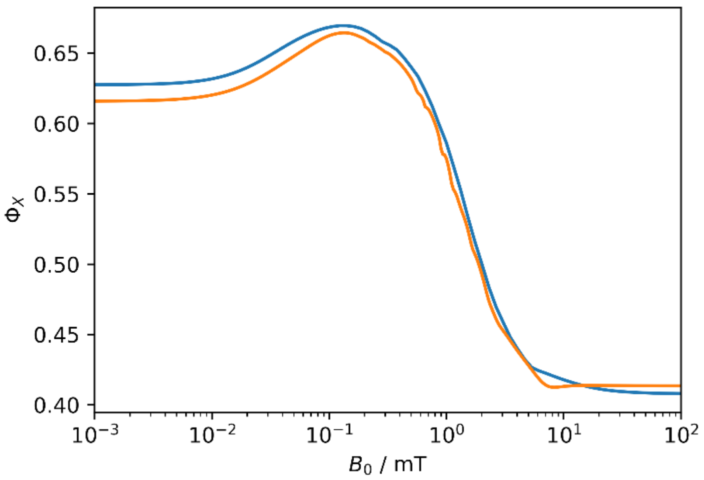

Fig A: Dependence of the singlet recombination yield Φ_X on the flux density of the applied magnetic field for the $FH^{\bullet}/O_2^{\bullet-}/A^{\bullet-}$ triad system with instantaneous spin relaxation in $O_2^{\bullet-}$. The two curves shown apply to models of different complexity: For the orange curve, 3 hyperfine coupled nuclei have been retained in the flavin semiquinone (N5, N10, H5) and one nucleus in the ascorbyl radical (H4); for the blue curve the model included 5 hyperfine coupled nuclei in the flavin semiquinone (N5, N10, H5, Hβ1, H6) and one in the ascorbyl radical (H4). The models provide comparable results, thereby demonstrating that the four-nuclear-spin model used in the main document is of sufficient complexity to derive a realistic picture of the magnetosensitivity of this system. $k_{\Sigma} = 1$ μs^{-1}, $k_X = 10$ μs^{-1}.

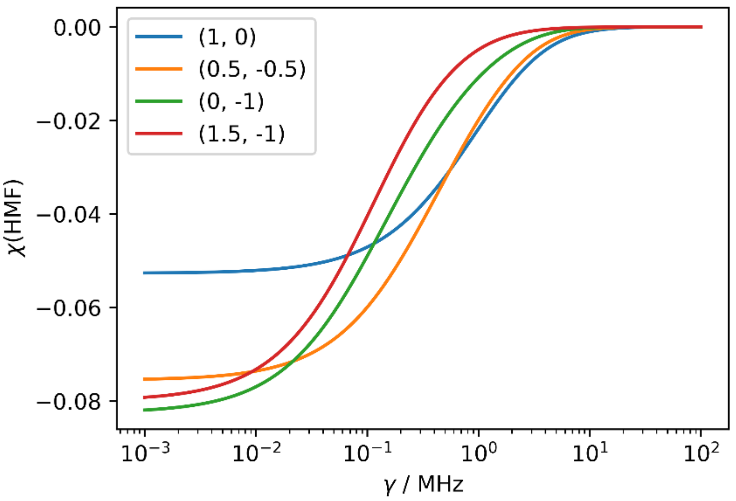

Fig B: Sensitivity of the hypomagnetic field effect of the $FH^{\bullet}/O_2^{\bullet-}/A^{\bullet-}$ triad system with instantaneous spin relaxation in $O_2^{\bullet-}$ to spin relaxation in the $FH^{\bullet}$ and $A^{\bullet-}$ radicals (rate: γ). The model assumed random-field relaxation, i.e., the master equation was of Lindblad form with six independent noise operators $\sqrt{\gamma}\,\hat{S}_{i,\alpha}$ ($i \in \{1,2\}$ and $\alpha \in \{x, y, z\}$). The model employed three nuclear spins for $FH^{\bullet}$ and one nuclear spin for $A^{\bullet-}$. Results are plotted for 4 chosen pairs of values of k_x and k_Σ, which are reported in the legend in terms of the decadic logarithm of their values in units of μs^{-1} ($k_x > k_\Sigma$).

Table B: Hypomagnetic field effects in $FH^\bullet/O_2^{\bullet-}/A^{\bullet-}$ triad system with instantaneous spin relaxation in $O_2^{\bullet-}$ calculated assuming absence (γ = 0) or presence (γ = 1 μs^{-1}) of spin relaxation in the $FH^\bullet$ and $A^{\bullet-}$ radicals (implemented as random-field relaxation) for selected values of k_X and k_Σ and models including 4 (N5, H5, N10 in $FH^\bullet$ and H4 in $A^{\bullet-}$) or 6 (additionally including H6 and Hβ1 in $FH^\bullet$) nuclear spins. Hypomagnetic field effects have been evaluated for a magnetic flux density of 0.29 μT relative to the geomagnetic field (with assumed flux density 55.26 μT) and are reported in percent. The data show that the simple model using the 4 largest hyperfine interactions provides an adequate description of the spin dynamics of this system. Inclusion of additional nuclear spins slightly attenuates the effects, but the same qualitative conclusions are manifest.

		4 nuclei		6 nuclei	
k_X / μs^{-1}	k_Σ / μs^{-1}	γ = 0	γ = 1 μs^{-1}	γ = 0	γ = 1 μs^{-1}
10	1	-5.27	-2.18	-4.96	-2.07
$10^{0.5}$	$10^{-0.5}$	-7.56	-1.99	-7.06	-1.88
1	0.1	-8.26	-1.09	-7.41	-1.02
$10^{1.5}$	0.1	-8.00	-0.49	-5.36	-0.42

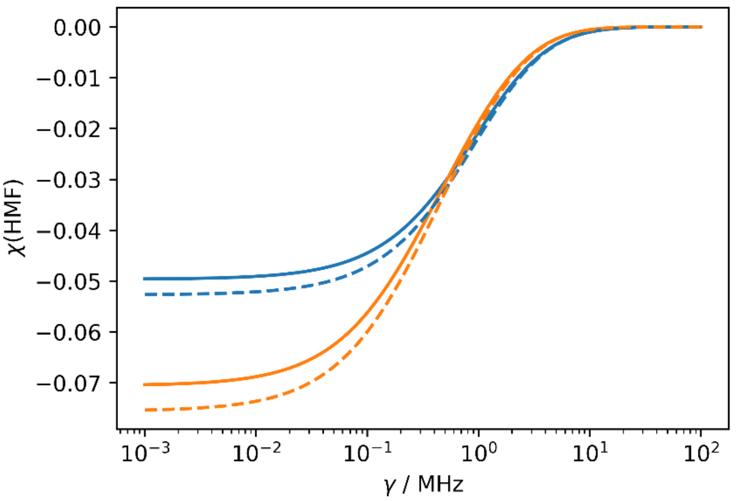

Fig C: The predicted hypomagnetic field effect (0.29 μT vs. 55 μT) for $FH^{\bullet}/O_2^{\bullet-}/A^{\bullet-}$ triad system with instantaneous spin relaxation in $O_2^{\bullet-}$ and variable spin relaxation in $FH^{\bullet}$ and $A^{\bullet-}$ (random-field relaxation at rate γ) for k_X = 10 μs^{-1} and k_Σ = 1 μs^{-1} (blue) and k_X = $10^{0.5}$ μs^{-1} and k_Σ = $10^{-0.5}$ μs^{-1} (orange) and for 4 (dashed) and 6 nuclear spins (solid lines). The simulations appear robust to enlarging the spin systems beyond the four core hyperfine interactions (N5, H5, N10 in $FH^{\bullet}$ and H4 in $A^{\bullet-}$) considered in the main document. For the simulations with 6 coupled nuclear spins, we have additionally included H6 and Hβ1 in $FH^{\bullet}$.

Code listing A: Python code to simulate isotropic and anisotropic magnetic field effects in radical pairs using tools form QuTip [2,3].

[2] J. R. Johansson, P. D. Nation, F. Nori: "QuTiP 2: A Python framework for the dynamics of open quantum systems", *Comp. Phys. Comm.* **184**, 1234 (2013).

[3] J. R. Johansson, P. D. Nation, F. Nori: "QuTiP: An open-source Python framework for the dynamics of open quantum systems", *Comp. Phys. Comm.* **183**, 1760–1772 (2012).

```
import numpy as np
from scipy import linalg
from scipy import integrate
from scipy import sparse
import qutip as qt
from matplotlib import pyplot as plt

opstr2fun = {'x': lambda dim: qt.spin_Jx((dim-1)/2),
             'y': lambda dim: qt.spin_Jy((dim-1)/2),
             'z': lambda dim: qt.spin_Jz((dim-1)/2),
             'p': lambda dim: qt.spin_Jp((dim-1)/2),
             'm': lambda dim: qt.spin_Jm((dim-1)/2),
             'i': qt.identity}
def mkSpinOp(dims, specs):
    ops = [qt.identity(d) for d in dims]
    for ind, opstr in specs:
        ops[ind] = ops[ind] * opstr2fun[opstr](dims[ind])
    return qt.tensor(ops)
def mkH1(dims, ind, parvec):
    axes = ['x', 'y', 'z']
    components = [v * mkSpinOp(dims, [(ind,ax)]) for v, ax in zip(parvec,
axes) if v!=0]
    if components:
        return sum(components)
    else:
        d = np.prod(dims)
        return qt.Qobj(sparse.csr_matrix((d, d), dtype=np.float64),
                       dims=[list(dims)]*2, type="oper", isherm=True)
def mkH12(dims, ind1, ind2, parmat):
    axes = ['x', 'y', 'z']
    components = []
    for i in range(3):
        for j in range(3):
            if parmat[i,j] != 0:
                components.append(parmat[i,j] * mkSpinOp(dims,
[(ind1,axes[i]), (ind2,axes[j])]))
    if components:
        return sum(components)
    else:
        d = np.prod(dims)
        return qt.Qobj(sparse.csr_matrix((d, d), dtype=np.float64),
                       dims=[list(dims)]*2, type="oper", isherm=True)

def singletYields(nucDims, indE, As, Omega0s, k0, kS, rho0=None):
    dims = np.concatenate(((2,2), nucDims))
    one = mkSpinOp(dims,[])
    Ps = 1/4 * one - mkH12(dims, 0, 1, np.identity(3))
    # Pt = one - Ps
    Hhfc = sum(mkH12(dims, indE[i], i+2, As[i]) for i in range(len(As)))
    if not rho0:
```

```
        rho0 = one/one.shape[0]
    print(np.trace(rho0))
    K = k0/2 * one + kS/2 * Ps
    yields = []
    Q = -np.array(rho0)
    for Omega0 in Omega0s:
        Hzee = mkH1(dims, 0, Omega0) + mkH1(dims, 1, Omega0)
        H0 = Hzee + Hhfc
        A = -1j * H0 - K
        x = linalg.solve_continuous_lyapunov(A.full(), Q)
        yields.append(kS*np.trace(x @ Ps.data).real)
    return np.array(yields)

def singletYieldsIso(nucDims, indE, aisos, omega0s, k0, kS, rho0=None):
    Omega0s = np.array([[0,0,1]]) * omega0s[:,np.newaxis]
    return singletYields(nucDims, indE, [np.eye(3)*a for a in aisos],
Omega0s, k0, kS, rho0=rho0)

def singletYieldsAvgAniso(nucDims, indE, As, omega0s, k0, kS, rho0=None):
    theta = np.linspace(0, np.pi/2, 101) # odd number of points!
    phi = np.linspace(0, 2*np.pi, 201)   # odd number of points!
    Theta, Phi = np.meshgrid(theta, phi, indexing='ij')
    oris = np.array([(np.cos(Phi)*np.sin(Theta)).reshape(-1),
                     (np.sin(Phi)*np.sin(Theta)).reshape(-1),
                     (np.cos(Theta)).reshape(-1)]).T
    avgYs = np.zeros(len(omega0s))
    for i, omega0 in enumerate(omega0s):
        Omega0s = omega0 * oris
        yields = singletYields(nucDims, indE, As, Omega0s, k0, kS,
rho0=rho0)
        avgYs[i] = integrate.simps(
                integrate.simps(yields.reshape(Theta.shape) *
np.sin(Theta), Theta[:,0], axis=0),
                Phi[0]) / (2*np.pi)
    return avgYs

def main():
    mT2angfreq = 9.274009994e-24/1.0545718e-34*2.00231930436256/1e9 #
Mrad/s/mT; ~28 MHz/mT

    # FH
    N5 = np.array([[-2.41368, -0.0662465, -0.971492],
                   [-0.0662465, -2.44657, 0.0485258],
                   [-0.971492, 0.0485258, 43.5125]]) * 2*np.pi
    N10 = np.array([[0.442319, 0.06085, 1.8016],
                    [0.06085, -0.0133137, -0.338064],
                    [1.8016, -0.338064, 23.1529]]) * 2*np.pi
    H5 = np.array([[-2.38856, 1.8683, 0.514044],
                   [1.8683, -40.6401, 0.0339364],
                   [0.514044, 0.0339364, -27.8618]]) * 2*np.pi
    # Asc
    H4 = 0.176 * mT2angfreq * np.eye(3)

    kS = 1.0 # 1/us
    k0 = 0.1 # 1/us
    Is = [1, 1, 0.5, 0.5]
    indE = [0, 0, 0, 1]
    nucDims = [round(2*I+1) for I in Is]

    b0 = np.logspace(-4, 3, 100) # mT
```

```
    ## isotropic
    hfcs = [np.trace(A)/3 for A in [N5, N10, H5, H4]]
    yields = singletYieldsIso(nucDims, indE, hfcs, b0*mT2angfreq, k0, kS)

    ## anisotropic
    # hfcs = [N5, N10, H5, H4]
    # yields = singletYieldsAvgAniso(nucDims, indE, hfcs, b0*mT2angfreq,
k0, kS)

    plt.semilogx(b0, yields)
    plt.show()

if __name__ == "__main__":
    main()
```
