# Supplementary material for: Radical triads, not pairs, may explain effects of hypomagnetic fields on neurogenesis
Source: PLoS Comput Biol. 2022 Sep 15;18(9):e1010519. doi: 10.1371/journal.pcbi.1010519 (PMC9514667; doi:10.1371/journal.pcbi.1010519)
Supplement: Attachment — Submitted filename: Response to reviewers.pdf [file pcbi.1010519.s007.pdf]

Reply to Reviewers

We thank the reviewers for their comments and suggestions, based on which we have been able to further improve our manuscript. Specifically, we have clarified the model in view of mobile radicals, expanded on the discussion of feasibility, and provided a detailed account on the infeasibility of flavin/ascorbyl radical pairs. Our point-by-point reply addresses all raised points in detail. The reviewers' comments are shown in blue followed by our reply in black.

Reviewer #1

The manuscript describes a theoretical study where a three-spin model comprising flavin, superoxide and ascorbyl radicals is shown to potentially account for observations from previous experimental studies where neurogenesis and hippocampus-dependent cognition adversely respond to hypomagnetic field exposure. The authors make a compelling case that this is a physically more realistic basis for these effects than another theoretical study [ref 4 in this manuscript] where only two spins are accounted for. I agree that it's difficult to see how the flavin semiquinone / superoxide RP proposed in [4] could be singlet born. It is also hard to justify neglecting the fast spin relaxation rates expected for superoxide, as is done in [4].

With one caveat (detailed below) I think the calculations presented seem reasonable and the implications of this study are interesting, not only in the context of neurogenesis etc, but also animal magnetoreception. The anisotropic nature of the calculated MF effects when flavin is treated as protein bound are compelling (and would likely be necessary for a compass sense), as are the possible implications of MF effects on ascorbic acid concentration in the context of magnetic signalling in neurons. Subject to the satisfactory answer to the points below, I therefore recommend this manuscript for publication.

We thank the reviewer for their assessment and valuable suggestion.

Main Point

I might be being thick, but I'm a bit confused about how diffusion and tumbling of $FH^{\bullet}$ are treated. On Page 7, the authors state: "...the mobile radicals $A^{\bullet-}$ and $O^{\bullet-}_2$ may escape the reactive encounter by diffusion out of the reaction site,...". Although not explicitly stated, the implication is that the $FH^{\bullet}$ is considered immobile by comparison; I guess because it is protein-bound? If no binding is assumed, then the diffusion of superoxide will be comparatively fast, but that of $FH^{\bullet}$ and $A^{\bullet-}$ will be of a similar order. In that case (as in Figs 2a and a, for example), an escape rate for $FH^{\bullet}$ should be included in K, which it isn't? or have I missed something? Elsewhere (page 9-10), the authors do discuss differences between protein-bound and free flavins, but in the context of the hyperfine couplings. Here, it is a bit clearer from the legend of Figure 2 that either only the isotropic contributions (for free flavin, panels a and b) or the entire anisotropic tensorial components are being accounted for. That said, in both contexts these things should be stated much more clearly than there are. The apparent lack of escape rate for $FH^{\bullet}$ should also be justified if indeed it is absent from the free flavin data and an explanation given for whether this is expected make any difference.

The reviewer raises an important point. The reaction scheme in Fig. 1b and the formulation of K does not include the escape of $FH^{\bullet}$, because $FH^{\bullet}$ has been considered immobile, i.e. protein-bound, as correctly assumed by the reviewer. We eventually apply the model to free radicals in the form of a micro-reactor model, which, as the reviewer again correctly suggests, formally necessitates the inclusion of the flavin escape as an additional processes. However, for the chosen formulation (in the employed limit of phi = 0), the predicted magnetic field effects are independent of the values of the individual escape rates (including the one for $FH^{\bullet}$) and only dependent on their sum (via k_{Σ}, which is the only parameter specified explicitly). Therefore, the results are correct regardless of how k_{Σ} is partitioned over the contributing rates of elementary processes (including or excluding the escape of $FH^{\bullet}$). We have considered including the additional escape rate explicitly, but eventually decided to not do so explicitly for succinctness while pointing out the assumption of an immobilized $FH^{\bullet}$. This is now accompanied by a statement on the differences (namely none on the level of the current formulation) arising from assuming a mobile $FH^{\bullet}$.

Specifically we now state the following in the Model section: "Here, we have assumed that $FH^\bullet$ is bound in the reaction site and thus does not escape (see Figure 1b). This is a natural choice for a protein-bound radical. A mobile $FH^\bullet$ could escape the encounter zone, thereby necessitating an additional escape rate (in terms of the used micro-reactor model). However, since the results as discussed below depend on the sum of escape rates only, we shall here spare us from explicitly introducing this additional rate constant for succinctness." In addition, we state in the caption to Fig. 1b that the scheme assumes an immobile, i.e. protein bound $FH^\bullet$.

Minor points

We thank the reviewer for their excellent suggestions, which we have accepted in their entirety.
Lines 13-14 – "…hypomagnetic fields (HMF; by means of near elimination of the geomagnetic field; 0.29 μT))". A bracket has been closed without having been opened.

The extraneous bracket was removed.

Lines 25-26 – "A recent theoretical study by the Simon-group [4]…" For the sake of delicacy, perhaps consider dropping direct references to the 'Simon group' here and elsewhere and simply refer to the publication in question?

We did not make the name explicit to blame, but to give due credit to Simon and group for highlighting the possibility of a radical-reaction-based explanation of the hypomagnetic field effect. While we do not think that their model is practically viable and needs to be extended, e.g. by introducing a third radical, as we do here, we still wanted to give due credit for initiating this field. However, we have followed the reviewer's advice as our intention could apparently be misinterpreted.

Lines 36-37 – "…and amongst each other, via exchange and dipolar interactions." Because this passage describes interactions between unpaired electron spins of a radical pair, perhaps it's more accurate to replace the word 'amongst' with 'between'.

Done.

Figure 1, legend – so the figure and its legend can stand alone, the various parameters (e.g., kf, kE) and notations (e.g., 1[…], DHA) should be defined briefly in the legend.

We now give brief explanations of the rates and abbreviations.

Lines 150-151 – "First, the primary FH•/O•−2 pair can react subject to further oxidation the FH•…" change to 'further oxidation of the FH•…'

Corrected.

Line 170 – presumably the first word of the line is 'respectively'?

Indeed.

Lines 173-174 – are both the words 'for' and 'in' required?

A "σ(t)" got lost, and was re-added.

Page 15 – the authors mention inter-radical interactions as a problem if the superoxide were bound in order to reduce its rotational correlation time. Binding in a protein environment would very likely be a result of H-bonding, which itself might introduce new hyperfine interactions, thus negating some of the benefits of having a hyperfine-free radical in the system?

Indeed, the immobilisation of the superoxide radical will likely result in additional hyperfine interactions. This is another argument against $FH^\bullet/O_2^{\bullet-}$ radical pairs, which would lose much of their superior magnetosensitivity in this scenario. We have added a brief note about this to the Introduction: "In addition, superoxide immobilisation

would likely introduce new hyperfine interactions, thus negating its core benefit of being devoid of hyperfine interactions and questioning models deriving superior magnetosensitivity from this property."

Lines 363-364 – Odd use of the use of the word 'eventually' in: "Eventually, note that the assumption that ascorbyl acts as scavenger radical is not critical…". Something like 'finally' might be better.

Changed to "finally".

Page 19 – the authors include an interesting discussion on the implications of possible MF effects on ascorbic acid concentrations in the context of its role as a neuromodulator of synaptic activity. I wonder if this might have relevance to other reports where flavin- and flavoprotein- dependent MF effects on neuronal activity have been reported (e.g., Sci. Rep. 2014, 4 : 5799; J. Neurosci. 2016, 36, 10742; bioRxiv 2021, DOI: 10.1101/2021.10.29.466426)?

The mentioned woks are prime examples of flavin-dependent magnetic field effects presumably detected via redox regulation of signal transduction pathways. It is not inconceivable that similar processes are at play in this context, including the involvement of the ascorbyl and associated potential neuromodulation. However, important difference exist, foremost the light-induction of the reaction. Specifically, it appears that here the three-radical model is not necessitated by the experimental observations so far (although it could still be relevant as an enhancing component) and that a model based on a radical pair, e.g. an intramolecular pair in FAD or a flavin/ascorbyl produced upon photo-reduction, might be sufficient (cf. reply to point 3 raised by Reviewer 3). We now cite the suggested references as examples of light-induced flavin-dependent MFEs on neuronal activity mediated via redox regulation of signal transduction pathways, but refrain from a discussion of details as this would necessitate extensive room and lead away from the system discussed here.

We have added the following: "On a broader scale, the intricate processes impacting on the ROS and ascorbic-acid balance discussed here could also apply to the light-dependent magnetic field effects on neuronal activity discussed in [Marley 2015, Giachello 2016, Bradlaugh 2021], which too are presumably detected via redox regulation of signal transduction pathways. In this case, a model based on a radical pair, e.g. an intramolecular pair formed in FAD or, maybe, a flavin/ascorbyl radical pair, produced upon photo-reduction appears to align with the experimental observations [Bradlaugh 2021]. Still, it is not inconceivable that R3M processes are at play in this context too."

Lines 529-430 – The following line feels like it's missing a clause: "In this situation, the magnetic isotope effect introduced by substituting naturally abundant oxygen by its 17O enriched isotopologues."

Corrected.

Reviewer #2

This manuscript proposes a new explanation of observations reported by Zhang et al. [2] that hippocampal neurogenesis and hippocampus-dependent cognition in mice differ in the geomagnetic field (55.26 μT) and in a hypomagnetic field (0.29 μT).

I completely agree with the authors' view that the interpretation of these observations put forward by Rishabh et al. [4] (via a flavin-superoxide radical pair reaction) is complete nonsense, although Ramsay and Kattnig are far too diplomatic to phrase it that strongly. Additional criticisms of Rishabh et al. include their neglect of radical-radical interactions and the ridiculously oversimplified hyperfine Hamiltonian they used to model magnetic field effects on the reactions of [$FH^{\bullet}$ $O_2^{\bullet -}$].

Although the R3M mechanism is speculative, as Ramsay and Kattnig openly acknowledge, it provides a much more plausible rationalisation of the rather surprising findings of Zhang et al. The model is clearly described and intelligently used to explore effects of hypomagnetic and geomagnetic fields on models of the flavin-superoxide-ascorbyl spin system in which the flavin radical undergoes either fast or slow rotational diffusion and the superoxide relaxes instantaneously.

We thank the reviewer for their assessment. We are glad that our model was able to convince and that it allows putting Rishabh et al.'s suggestion of the effect's origin on a more tenable, concrete foundation.

While I have no doubt that this manuscript should be accepted, I think it would benefit from a few caveats.

1. On the basis of their simulations, Ramsay and Kattnig claim that a magnetic field of only 1 μT "can elicit an effect of the order of 1%" (lines 288-289) or "larger than 1%" (line 441). If true in practice, this would be truly remarkable. However, I wonder about the assumption that spin relaxation in the flavin and ascorbyl radicals is negligibly slow on the timescale of k_{Σ}^{-1} = 10 μs. Also, is it really acceptable to omit dipolar interactions which, as Kattnig has previously argued [20], can greatly attenuate the sensitivity of radical pairs to weak magnetic fields?

We thank the reviewer for their cautious caveat. The quoted statements describe the results as presented in Fig. 2 and 3. These have been realized under the assumption that the characteristic time of spin relaxation exceeds the lifetime of the radical triad. Indeed, in practice, this condition might not be met for the lifetime of 10 μs underpinning the statement, given that typical relaxation times of flavin and ascorbyl radicals are of the order of 1 μs, as discussed in the model section. In order not to mislead the reader about the nature of our optimistic estimate, we now highlight the assumption of slow spin relaxation relative to the lifetime and state results for k_{Σ} = 1 μs^{-1} for comparison, for which a 1 % effect requires 12 μT under comparable conditions.

Specifically now state the following: "Selected parameter values of k_X and k_{Σ} can in principle lead to sensitivity to remarkably low fields. For example, for k_X = 1 μs^{-1} and k_{Σ} = 0.1 μs^{-1} (blue lines in Fig. 3), the field of only 1 μT is predicted to elicit an effect of the order of 1 %. Note, however, that this optimistic result is conditioned on the assumption of slow spin relaxation relative to the triad lifetime. For a lifetime comparable to typical spin relaxation times of flavin and ascorbyl radicals (k_{Σ} = 1 μs^{-1}, k_X = 10 μs^{-1}), 12 μT are estimated to be required for an effect of 1%. Overall, this nonetheless suggests that hypomagnetic field effects could be more pronounced and more interesting to study than anticipated."

Concerning the electron-electron dipolar interactions, we argue that the effect can be neglected in first order. First, observe that reference [20] discusses the (detrimental) effect of the electron-electron dipolar coupling on the magnetosensitivity of immobilized radical systems. This is of no concern here as for the considered model at least two of the radicals are mobile as a result of which the mutual dipolar coupling of the system is averaged to zero by the relative molecular motion. It is true that in this scenario the dipolar coupling can still induce spin relaxation if the radical concentration is large. However, for typical parameters, e.g. a diffusion coefficient of D = 100 Å^2/ns, an assumed particle radius of 3 Å, and the three radicals occupying an effective volume corresponding to a sphere of 10 nm radius, the associated relaxation times are estimated to be long relative to the intrinsic relaxation rates of the radicals (i.e. 6 μs according to eq. 115 in Abragam's Principles of Nuclear Magnetism). Thus, with the focus on realistic spin relaxation time as introduced above, the electron-electron dipolar coupling is not expected to further attenuate the magnetic field effects. We nonetheless added a brief remark in the Model section: "Note that we neglect the electron-electron dipolar interaction, as at least one constituent of each pair of radicals is assumed to be mobile, thereby averaging the interaction to zero by the mutual diffusive motion."

2. Although the R3M mechanism is now 5 years old [26] and, unlike Rishabh et al., is grounded in solid science, there is still, as far as I know, no direct experimental evidence for a chemical R3M magnetic field effect. In the interests of complete transparency, and to encourage others to seek experimental proof of the mechanism, I think it would be helpful if the authors could mention this fact.

We are not aware of ongoing endeavours to identify R3M-effects in experiments. Unfortunately, only a small number of groups have the technical possibilities and access to friable experimental system, such as cryptochromes, and these groups, apparently, find enough unresolved questions in the realm of the RPM. We have added a statement to the Introduction and the Conclusions to encourage experimental investigations of the effect. In the Introduction we state: "Frustratingly, the experimental confirmation of these three-radical effects is still outstanding five years after their suggestion." In the Conclusions we urge: "We also hope that this work will entice the experimental investigation of magnetic field effects due to three-radical correlation."

3. The manuscript ends with the "exciting prospect of manipulating neuronal processes by applied static and oscillatory fields via direct modulation of ROS levels" (lines 465-467) and the speculation that "redox homeostasis

could be regarded as an indirect/coincidental quantum effect in biology" (lines 469-470). A recent review article on quantum biology*, of which Dr Kattnig was an author, contained the following statement towards the end of the section on "Magnetic Field Effects on Spin-Dependent Reactions in Biology":

> *As we have discussed here, there are stringent requirements that must be fulfilled to elicit magnetic-sensitivity on spin-selective reactions (Figure 6). The likelihood of all the necessary conditions being met in any one biological system appears to be low and are perhaps easier to realize in diffusing systems than immobilized radicals. While accidental magnetic-sensitivity cannot be ruled out per se, it seems possible that protein magnetoreceptors, should they truly exist and be physiologically relevant, might need to have been evolutionally optimized for large MFEs to manifest.*

This passage, which may even have been written by Dr Kattnig, seems pretty accurate to me. Bearing in mind the additional "complexity" of the R3M process (lines 342-343), wouldn't an "indirect/coincidental" R3M effect in biology have even more "stringent requirements" than a RPM effect? Perhaps the authors could add a short discussion of the likelihood of accidental R3M magnetic sensitivity?

*Kim et al. Quantum biology: an update and perspective. Quantum Rep. 2021, 3, 1-48.

Touché. It is true that the suggested process is subject to stringent requirements. While such requirements are clearly more plausibly realizable if underpinning a functional trait, such as magnetoreception, a random magnetosensitivity cannot be rule out. In fact, it might have to be even expected despite small intrinsic probabilities as a consequence of the omnipresence of radical processes in biology. With view of the quoted paragraph, the suggested process involves diffusing systems, for which the necessary prerequisites are arguable easier to fulfil. Furthermore, additional factors, such as the temporal separation of the superoxide/flavin semiquinone reaction and ascorbyl radical encounter, have already been discussed in the "Thoughts on the viability of three-radical processes" section, which together suggest that the process is not too disparate from a radical-pair process. In any case, we have added an additional paragraph to the Discussion which tries to address the question. Obviously, no concrete answer can be provided here, nor in the "easier" context of radical-pair magnetoreception. Nonetheless, we hope that the discussion will stimulate further development and thoughts:

"Previous works have expressed the opinion that protein magnetoreceptors, should they truly exist, might need to have been evolutionally optimized for large MFEs to manifest [Kim 2021]. Bearing in mind the additional complexity of the proposed process, this suggests that a priori the likelihood of radical-recombination based magnetosensitivity in neurogenesis is small. This argument is somewhat extenuated here as the stringent requirements are arguably [Kim 2021] easier to realize in the implied diffusing systems than immobilized radicals, as discussed above. It is furthermore tempting to speculate that the three-radical processes has in fact been evolutionally optimized, but with a different objective in mind. For example, it is not inconceivable that a three-radical process has evolved optimally to, for example, inhibit excessive efflux of toxic superoxide in the flavin reoxidation in the constant geomagnetic field. As a byproduct, this might have led to a dormant magnetic field sensitivity, which only becomes apparent under non-physiological conditions, as is hypomagnetic field exposure. Finally, even a coincidental R3M effect in biology cannot be ruled out, as stringent requirements are opposed by the sheer number of possible radical recombination reactions associated with the generation and dissipation of reactive oxygen species."

Minor issues

We have implemented all listed minor issues as listed below and thank the reviewer, thirteen times, for their advertence in assessing our manuscript and helpful suggestions.

Abstract, 4th line: **theoretical** study (?)

Page 5, line 114: ascorbyl not ascrobyl

Page 6, Fig. 1: I don't think the abbreviation DHA is explained anywhere

Page 7, lines 150-151: further oxidation **of** the

Page 8, lines 167-168: I think “zeros coherences” would be clearer as “destroys coherences” (or similar). This would avoid possible confusion with zero (quantum) coherences.

Page 8, line 173: equation of motion for $\hat{\sigma}(t)$ (?)

Page 9, line 184: yield the superoxide **radical**

Page 13, line 278: magnetosensitive not magnetosenstive

Page 15, line 300: spin relaxation **is** excluded

Page 15, line 315: the **molecules’** tumbling motion

Page 16, line 346: same for **triplet**

Page 19, line 418: inconceivable not unconceivable

Page 19, lines 429-430: The sentence, “In this situation, the magnetic isotope effect introduced by substituting naturally abundant oxygen by its ^{17}O enriched isotopologues”, seems incomplete. Something missing?

All points have been implemented as suggested.

Reviewer #3

Experimental effects of hypomagnetic magnetic fields on adult hippocampal neurogenesis and hippocampus-dependent cognition in mice have been reported. A recent theoretical study has proposed the radical pair mechanism, leveraging a singlet born flavin-superoxide radical pair to explain these experimental observations. This manuscript, reiterates the criticisms of this model presented in another recent work, which suggests that there are two major problems (very fast superoxide radical spin relaxation and the unreasonable (singlet) assignment of the initial spin-state of the radical pair), and proposes an alternative mechanism based on the radical triad model, previously developed by one of the authors and in previous publications proposed as an alternative to the RPM for explaining the avian magnetic compass.

The manuscript itself is clear and very well written. The model itself is already well established and this work applies it to the specific case of reduced ROS concentrations in hypomagnetic fields for initially flavin-superoxide radical pairs subject to scavenging from ascorbate anion radicals. The results of the simulations are convincing and the paper goes to appropriate lengths to justify the conditions that might lead to these very specific three radical systems being formed. I believe that the authors successfully argue that this is a much more plausible mechanism than the flavin-superoxide radical pair, but I think they need to address possible alternative mechanisms with similarities to their proposal which do not require a three radical intermediate. I think that overall the paper is worth publishing PLOS computational biology, but I would like the authors to address the following three points before the article can be accepted for publication:

We thank the reviewer for their assessment and suggestions. We are not aware of the other recent work that apparently has expressed similar criticism, except for a pre-print of our own manuscript posed to arXiv prior to submission. We also note that Simon and co-workers have already mentioned the ostensible weaknesses of their radical-pair model, albeit without offering a resolution.

1) The authors argue that ascorbate anion radical is only an example possible scavenger radical and imply that other scavenger radicals are possible alternatives. I think that this is somewhat disingenuous. As the authors clearly describe, the fast relaxation of the superoxide essentially leads to the triad behaving as an effective flavin-

ascorbate radical pair (the reduced Liouvillian only has coherent terms from this pair). They also highlight that the very substantial low field effects observed in the simulations are a direct result of the particular hyperfine structure of the ascorbate anion radical and that large LFEs have been observed experimentally for flavin-ascorbate radical pairs. Therefore, for most possible alternative scavenger radicals, the LFE and thus hypomagnetic effect will be substantially less, and for many cases will be negligible. I think the authors need to address this point a little more straightforwardly. (Right now it feels like they are hedging their bets by using ascorbate for its great properties but defending against arguments that ascorbate might not be appropriate in this biological context).

We fully agree with the reviewer, but reject disingenuity. Our intention was to merely point out that other radicals are in principle imaginable in the role of the scavenger. This may or may not give rise to lower magnetosensitivity due to less ideal hyperfine structures, but this is a secondary question, which does not affect the principle possibility of a different radical scavenger. In fact, and again in principle, our wording was chosen to even encompass the ominous $Z^{\bullet}$ radical popular with magnetoreception (i.e. a hypothetical slowly relaxing radical devoid of hyperfine interactions), for which an additional boost of magnetosensitivity is expected. Having said that, we agree with the reviewer that the ascorbyl radical appears to be an ideal and realistic choice in many respects. We thus more straightforwardly point out the beneficial properties of the ascorbyl radical and explicitly state that, while in principle imaginable, other radicals might give rise to lower performance, in particular if their hyperfine structure is more complex. We are not aware of any arguments that preclude ascorbyl in the biological context. On the opposite, it seems to be a very appropriate radical partner in particular in neuronal cells.

Thus, instead of the sentence "While the identity of this additional radical is not crucial, we consider the ascrobyl radical ($A^{\bullet -}$, monodehydroascorbic acid) in this role, predominantly for concreteness, but also inspired by the abundance of ascorbic acid in neurons and its established function [CovarrubiasPinto 2015]."
we now write:
"Here we consider the ascrobyl radical ($A^{\bullet -}$, monodehydroascorbic acid) in this role, predominantly for concreteness, but also inspired by the abundance of ascorbic acid in neurons, its established function [CovarrubiasPinto2015], and its favourable hyperfine structure, which is known to give rise to a large low field sensitivity [Lee 2014, Evans 2016]."

Furthermore, instead of "Eventually, note that the assumption that ascorbyl acts as scavenger radical is not critical (while a convincing case in favour of ascorbyl can be made based on the arguments from above)."
we now write:
"Finally, we note that other radicals than the ascorbyl radical could act as scavenger radicals (although convincing case in favour of ascorbyl can be made based on the arguments from above and its desirable hyperfine structure)."

2) Ascorbate anion is a known quencher of singlet oxygen. There have been a number of studies reporting various rate coefficients for the quenching reaction that vary quite substantially, but there seems to be a consensus about the reaction of superoxide with ascorbate ion by hydrogen atom abstraction (see for example https://doi.org/10.1016/j.jece.2022.107736). The authors make the case for substantial ascorbate concentrations, and so need to make the case for why the ascorbate anion (which will be at much higher concentrations than the ascorbate radical) does not quench the superoxide.

The reviewer makes an important point. As the concentration of the ascorbate will be significantly higher than that of the ascrobyl radical, the three radical processes will necessarily proceed in an environment abundant with ascorbate. We argue that the reaction of the ascorbyl radical with superoxide will still dominate the reactive encounter over the competing scavenging of the superoxide by ascorbate, because of the vastly larger (by four orders of magnitude) rate constant of the radical-radical reaction. Specifically, the ascorbate radical is known to react with $HOO^{\bullet}$ and $O_2^{\bullet -}$ at rates of $5x10^9$ $M^{-1}s^{-1}$ and $2.6x10^8$ $M^{-1}s^{-1}$, respectively, whereas a rate constant of $5x10^4$ $M^{-1}s^{-1}$ has been suggested for the reaction between AH^- and $O_2^{\bullet -}$ in ref [1]. This older measurement is in agreement with the more recent work suggested by the reviewer, which quotes 3.3×10^4 $M^{-1}s^{-1}$ for the latter reaction under physiological conditions. Tu et al. [2] have provided a theoretical explanation of the fast $O_2^{\bullet -}$/ascorbyl radical reaction in terms of the formation of an adduct between superoxide and ascorbate radical, followed by the addition of two protons to form a neutral intermediate, subsequent cyclisation and finally dissociating to DHA and H_2O_2. For the spin dynamics only the first addition, for which the quoted $2.6x10^8$ $M^{-1}s^{-1}$ rate constant provides a

lower bound, is relevant. This suggests that the radical-radical process could be sustained as the dominant reaction pathway in an environment rich in adsorbate. We also want to point out that even a small k_X' would not alter the validity of the model and derived conclusions provided that k_F is fast and the superoxide radical efficiently consumed in the reactive processes.

[1] D. E. Cabelli and B. H. J. Bielski, Kinetics and mechanism for the oxidation of ascorbic acid/ascorbate by HO_2/O_2^- (hydroperoxyl/superoxide) radicals. A pulse radiolysis and stopped-flow photolysis study, J. Phys. Chem., 1983, 87, 1809 —1812.

[2] Yi-Jung Tu, David Njus, H. Bernhard Schlegel, A theoretical study of ascorbic acid oxidation and $HOO^{\cdot}/O2^{\cdot-}$ radical scavenging, Org. Biomol. Chem., 2017,15, 4417-4431.

We have now added the following statement: "It is also interesting to note that the ascorbyl radical is more reactive to $HOO^{\bullet}$ and $O_2^{\bullet-}$ (reacting at rates of of $5x10^9$ $M^{-1}s^{-1}$ and $2.6x10^8$ $M^{-1}s^{-1}$, respectively) than the ascorbate ion (rate constants of the order $5x10^4$ $M^{-1}s^{-1}$) [Cabelli 1983, Ruiyang 2022, Njus 2020, Tu 2017]. This suggests that the radical-radical process could be sustained as the dominant reaction pathway in an environment rich in adsorbate."

3) Based on points made in 1) and 2) above, the authors argue that the ascorbate radical is present at sufficient concentrations for their model, and that the magnetic field sensitivity arises from essentially a flavin-ascorbate radical pair. This being the case, they need to address why the triad model is necessary. If flavin reacts with superoxide to make flavin-superoxide radical pairs, this is the source of superoxide. To continue producing superoxide, the flavin radical must be reoxidised. Thus an f-pair formed of flavin radical and ascorbate radical could regenerate the oxidised form of the flavin in a magnetically-sensitive process. Thus the ground state flavin concentration could change with the application of a hypomagnetic field and this would be reflected in the resulting concentration of superoxide radicals. Such a mechanism has the same 'active' radical pair as the triad model while not requiring the formation of a radical triad. The authors should address this similar yet simpler alternative mechanism and demonstrate why the triad model is more likely.

In the limit of instantaneous spin relaxation, the two models are conceptually similar, yet the reaction outcomes are very different. Specifically, the reaction scheme as suggested by the reviewer would imply an increase in the superoxide concentration in the hypomagnetic field, whereas the three radical encounter gave rise to a reduction. Specifically, the recombination of the flavin semiquinone/ascorbyl radical pair encountering as f-pair will be impeded in the hypomagnetic field (in line with the low-field effect). This will give rise to an increase in the semiquinone concentration which is to react (in part) with molecular oxygen to increase the superoxide concentration. On the other hand, in the three radical model in the assumption of efficient superoxide reactivity, superoxide will only be released if the scavenging processes was efficient, which again is impeded by the hypomagnetic field, thus reducing superoxide concentration for the zero-field. Thus the three radical picture allows to construct a scenario that naturally gives rise to the observed sign of the magnetic field effect. The difference of the two models boils down to reactivity and conditions of the encounter processes, i.e. reaction dynamics. While the three-radical model assumes reactive superoxide and an early encounter with the ascorbyl radical, the $FH^{\bullet}/A^{\bullet-}$-radical pair model assumes low reactivity and a late encounter with ascorbyl. Consequently, changes in reaction condition could bring about qualitatively very different magnetic field effects. We hesitate to claim that one scenario is more likely than the other, but the explanation of the observed MFEs within the accepted assumptions about the reactivity and chemistry of the involved radicals appears to require the three-radical paradigm.

We have added the following paragraph as the end of section 'Benefits of the three-radical model' after line 355: "Note that the model based on the active flavin semiquinone and ascorbyl radical pair alone could not explain the hypomagnetic field effect. Specifically, the recombination of the flavin semiquinone/ascorbyl radical pair encountering as f-pair will be impeded in the hypomagnetic field (in line with the low-field effect). This will give rise to an increase in the free semiquinone concentration which is to react (in part) with molecular oxygen to increase the superoxide concentration."
