# Supplementary material for: Radical triads, not pairs, may explain effects of hypomagnetic fields on neurogenesis
Source: PLoS Comput Biol. 2022 Sep 15;18(9):e1010519. doi: 10.1371/journal.pcbi.1010519 (PMC9514667; doi:10.1371/journal.pcbi.1010519)
Supplement: Attachment — Submitted filename: Ramsay Kattnig PLoSComputBiol review.docx [file pcbi.1010519.s006.docx]

## Radical triads, not pairs, may explain effects of hypomagnetic fields on neurogenesis

## Ramsay and Kattnig

This manuscript proposes a new explanation of observations reported by Zhang et al. [2] that hippocampal neurogenesis and hippocampus-dependent cognition in mice differ in the geomagnetic field (55.26 μT) and in a hypomagnetic field (0.29 μT).

I completely agree with the authors’ view that the interpretation of these observations put forward by Rishabh et al. [4] (via a flavin-superoxide radical pair reaction) is complete nonsense, although Ramsay and Kattnig are far too diplomatic to phrase it that strongly. Additional criticisms of Rishabh et al. include their neglect of radical-radical interactions and the ridiculously oversimplified hyperfine Hamiltonian they used to model magnetic field effects on the reactions of $[\text{FH}^{\bullet} \text{O}_{2}^{\bullet-}]$.

Although the R3M mechanism is speculative, as Ramsay and Kattnig openly acknowledge, it provides a much more plausible rationalisation of the rather surprising findings of Zhang et al. The model is clearly described and intelligently used to explore effects of hypomagnetic and geomagnetic fields on models of the flavin-superoxide-ascorbyl spin system in which the flavin radical undergoes either fast or slow rotational diffusion and the superoxide relaxes instantaneously.

While I have no doubt that this manuscript should be accepted, I think it would benefit from a few caveats.

1. On the basis of their simulations, Ramsay and Kattnig claim that a magnetic field of only 1 μT “can elicit an effect of the order of 1%” (lines 288-289) or “larger than 1%” (line 441). If true in practice, this would be truly remarkable. However, I wonder about the assumption that spin relaxation in the flavin and ascorbyl radicals is negligibly slow on the timescale of $k_{\Sigma}^{-1}$ = 10 μs. Also, is it really acceptable to omit dipolar interactions which, as Kattnig has previously argued [20], can greatly attenuate the sensitivity of radical pairs to weak magnetic fields?

2. Although the R3M mechanism is now 5 years old [26] and, unlike Rishabh et al., is grounded in solid science, there is still, as far as I know, no direct experimental evidence for a chemical R3M magnetic field effect. In the interests of complete transparency, and to encourage others to seek experimental proof of the mechanism, I think it would be helpful if the authors could mention this fact.

3. The manuscript ends with the “exciting prospect of manipulating neuronal processes by applied static and oscillatory fields via direct modulation of ROS levels” (lines 465-467) and the speculation that “redox homeostasis could be regarded as an indirect/coincidental quantum effect in biology” (lines 469-470). A recent review article on quantum biology*, of which Dr Kattnig was an author, contained the following statement towards the end of the section on “Magnetic Field Effects on Spin-Dependent Reactions in Biology”:

*As we have discussed here, there are stringent requirements that must be fulfilled to elicit magnetic-sensitivity on spin-selective reactions (Figure 6). The likelihood of all the necessary conditions being met in any one biological system appears to be low and are perhaps easier to realize in diffusing systems than immobilized radicals. While accidental magnetic-sensitivity cannot be ruled out per se, it seems possible that protein magnetoreceptors, should they truly exist and be physiologically relevant, might need to have been evolutionally optimized for large MFEs to manifest.*

This passage, which may even have been written by Dr Kattnig, seems pretty accurate to me. Bearing in mind the additional “complexity” of the R3M process (lines 342-343), wouldn’t an “indirect/coincidental” R3M effect in biology have even more “stringent requirements” than a RPM effect? Perhaps the authors could add a short discussion of the likelihood of accidental R3M magnetic sensitivity?

*Kim et al. Quantum biology: an update and perspective. Quantum Rep. 2021, 3, 1-48.

**Minor issues**

Abstract, 4th line: **theoretical** study (?)

Page 5, line 114: ascorbyl not ascrobyl

Page 6, Fig. 1: I don’t think the abbreviation DHA is explained anywhere

Page 7, lines 150-151: further oxidation **of** the

Page 8, lines 167-168: I think “zeros coherences” would be clearer as “destroys coherences” (or similar). This would avoid possible confusion with zero (quantum) coherences.

Page 8, line 173: equation of motion for $\hat{\sigma}(t)$ (?)

Page 9, line 184: yield the superoxide **radical**

Page 13, line 278: magnetosensitive not magnetosenstive

Page 15, line 300: spin relaxation **is** excluded

Page 15, line 315: the **molecules’** tumbling motion

Page 16, line 346: same for **triplet**

Page 19, line 418: inconceivable not unconceivable

Page 19, lines 429-430: The sentence, “In this situation, the magnetic isotope effect introduced by substituting naturally abundant oxygen by its ^17^O enriched isotopologues”, seems incomplete. Something missing?
